# Financial Counseling Is Associated with Reduced Financial Difficulty Scores in Head and Neck Cancer Patients Treated with Radiation Therapy

**DOI:** 10.3390/cancers13112516

**Published:** 2021-05-21

**Authors:** Mark Farrugia, Han Yu, Sung Jun Ma, Austin J. Iovoli, Kayleigh Erickson, Elizabeth Wendel, Kristopher Attwood, Kimberly E. Wooten, Vishal Gupta, Ryan P. McSpadden, Moni A. Kuriakose, Michael R. Markiewicz, Jon M. Chan, Wesley L. Hicks, Mary E. Platek, Andrew D. Ray, Elizabeth A. Repasky, Anurag K. Singh

**Affiliations:** 1 Department of Radiation Medicine, Roswell Park Comprehensive Cancer Center, 665 Elm Street, Buffalo, NY 14203, USA; Mark.Farrugia@roswellpark.org (M.F.); SungJun.Ma@RoswellPark.org (S.J.M.); Austin.Iovoli@RoswellPark.org (A.J.I.); Mary.Platek@RoswellPark.org (M.E.P.); 2 Jacobs School of Medicine and Biomedical Sciences, University at Buffalo, The State University of New York, 955 Main Street, Buffalo, NY 14203, USA; 3 Department of Biostatistics and Bioinformatics, Roswell Park Comprehensive Cancer Center, 665 Elm Street, Buffalo, NY 14203, USA; Han.Yu@RoswellPark.org (H.Y.); Kristopher.Attwood@RoswellPark.org (K.A.); 4 Department of Cancer Prevention and Control, Roswell Park Comprehensive Cancer Center, 665 Elm Street, Buffalo, NY 14203, USA; Kayleigh.Erickson@RoswellPark.org (K.E.); Elizabeth.Wendel@RoswellPark.org (E.W.); Andrew.Ray@RoswellPark.org (A.D.R.); 5 Department of Head and Neck Surgery, Roswell Park Comprehensive Cancer Center, 665 Elm Street, Buffalo, NY 14203, USA; Kimberly.Wooten@RoswellPark.org (K.E.W.); Vishal.Gupta@RoswellPark.org (V.G.); Ryan.McSpadden@RoswellPark.org (R.P.M.); Moni.Kuriakose@RoswellPark.org (M.A.K.); Michael.Markiewicz@RoswellPark.org (M.R.M.); Jon.Chan@RoswellPark.org (J.M.C.); Wesley.Hicks@RoswellPark.org (W.L.H.J.); 6 Department of Oral and Maxillofacial Surgery, School of Dental Medicine, University at Buffalo, The State University of New York, 3435 Main Street, Buffalo, NY 14214, USA; 7 Department of Neurosurgery, Jacobs School of Medicine and Biomedical Sciences, University at Buffalo, The State University of New York, 955 Main Street, Buffalo, NY 14203, USA; 8 Department of Dietetics, D’Youville College, 270 Porter Avenue, Buffalo, NY 14201, USA; 9 Department of Immunology, Roswell Park Comprehensive Cancer Center, 665 Elm Street, Buffalo, NY 14203, USA; Elizabeth.Repasky@roswellpark.org

**Keywords:** financial counseling, financial toxicity, head and neck cancer

## Abstract

**Simple Summary:**

Financial toxicity (FT) can be devastating to cancer patients, and solutions are urgently needed. We investigated how providing financial counseling (FC) for head and neck cancer (HNC) patients undergoing radiation therapy impacted patients’ financial difficulties at the end of treatment. Beginning in July 2018, a dedicated financial counselor was provided, and all eligible patients received FC. Via a survey, patients who did not have FC reported a significant increase in financial difficulty at the end of treatment however those who had received FC did not report such an increase. Furthermore, in a statistical model FC was associated with significantly lower financial difficulty scores. Based on the findings, the employment of a financial counselor may be a viable, hospital-based approach to begin to address FT in HNC.

**Abstract:**

Background: Financial toxicity (FT) can be devastating to cancer patients, and solutions are urgently needed. We investigated the impact of financial counseling (FC) on FT in head and neck cancer (HNC) patients. Methods: Via a single-institution database, we reviewed the charts of HNC patients who underwent definitive or post-operative radiotherapy, from October 2013 to December 2020. Of these patients, 387 had provided baseline and post-treatment information regarding financial difficulty. In July 2018, a dedicated financial counselor was provided for radiation therapy patients and we subsequently examined the impact of FC on financial difficulty scores. Results: Following the hiring of a dedicated financial counselor, there was a significant increase in the proportion of patients receiving FC (5.3% vs. 62.7%, *p* < 0.0001). Compared with baseline scores, patients who did not undergo FC had a significant increase in reported financial difficulty at the end of treatment (*p* = 0.002). On the other hand, there was no difference in pre- and post-treatment scores in patients who had received FC (*p* = 0.588). After adjusting for gender and nodal status with a multiple linear regression model, FC was significantly associated with change in financial difficulty (β = −0.204 ± 0.096, *p* = 0.035). On average, patients who received FC had a 0.2 units lower change in financial difficulty score as compared with those with the same gender and nodal stage but without FC. Conclusions: Providing a dedicated financial counselor significantly increased the proportion of HNC receiving FC, resulting in the stabilization of financial difficulty scores post-treatment. Based on a multiple linear regression model, FC was independently associated with reduced financial difficulty. The employment of a financial counselor may be a viable, hospital-based approach to begin to address FT in HNC.

## 1. Introduction

“Problems a patient has related to the cost of medical care” are defined as financial toxicity (FT) by the National Cancer Institute. Furthermore, FT is also referred to as economic burden, economic hardship, financial burden, financial distress, financial hardship, and financial stress [1]. FT experienced by cancer patients continues to worsen as healthcare costs increase [2,3,4].

Head and neck cancer (HNC) patients are at particularly high risk for FT as they often require multi-modal therapy, have more out-of-pocket costs, have an increased reliance on cost-coping strategies, and are more likely to suffer production losses as a result of their disease and treatment [5,6,7,8]. Consequently, increased financial difficulties in HNC has been linked to impaired quality of life (QOL), increased missed appointments, reduced compliance with medications, and worse medical outcomes including survival [9,10,11]. Therefore, urgent mitigation strategies for FT among HNC patients are needed.

Reducing treatment costs will require efforts at the national, commercial, institutional, and physician-levels [4] Multiple physician societies have led initiatives to implement cost-effective strategies within clinical guidelines [12,13,14]. While the appropriate use of cost-effective treatment practices can certainly play a role, this approach alone cannot fully mitigate FT in HNC.

Financial counseling (FC) is a non-treatment-based intervention, which may reduce FT in HNC. In a national survey of cancer centers, 70.2% of oncologists stated they were reluctant to discuss cost of treatment with patients. Furthermore, while 96.5% of these centers offered discounts or assistance regarding drug pricing, only 54.4% offered discussions regarding cost of treatment [15]. Financial counselors can help patients navigate and provide transparency on benefits, expected costs, and cost-mitigating strategies [15]. Despite this, there is a paucity of data examining FC and FT. In this retrospective review, we assessed the impact of standard of care FC on FT in HNC patients undergoing definitive radiation therapy.

## 2. Materials and Methods

### 2.1. Patient Population

With a waiver of consent under approval from the Roswell Park Comprehensive Cancer Center Institutional Review Board for human subject protection (EDR-103707), the charts of HNC patients who underwent definitive or post-operative radiotherapy from October 2013 to December 2020 were reviewed. Of these patients, 408 had provided baseline information regarding financial difficulty. Of these patients, 387 had also completed a survey at the end of treatment.

### 2.2. Patient Data

Pertinent demographic and clinical data were recorded via chart review. Treatment details have previously been described [16,17,18]. Unless specifically noted, missing information comprised less than 1% for each variable. Marital status was documented at the time of treatment. Human papilloma virus (HPV) status was determined by p16 positivity. Staging was performed as per the American Joint Commission on Cancer, 7th edition. Insurance provider information was obtained via the financial counselor. Private insurance included any non-governmental or commercial plans. In instances where a patient had two different insurances, reporting priority was as follows: (1) private, (2) Medicare, and (3) Medicaid. Financial assistance was documented by the financial counselor and defined as institutional assistance with payment for radiation treatment.

### 2.3. Financial Counseling

In July 2018, a dedicated financial counselor was provided for radiation therapy patients. A letter describing benefits, options available to manage expenses, and anticipated expenses by both the patient and their insurance provider was mailed prior to treatment (Supplemental document 1). This letter was not sent in the following circumstances: (i) radiation consult and simulation occurred while patient was hospitalized; (ii) patient had out-of-network plans where we did not have access to fee schedules; and (iii) select patients with dual insurances where coordination of benefits could not be determined. Prior to hiring a dedicated financial counselor, FC was not readily available and only offered at patient request. Notably, cost-estimations were only for radiation therapy and not for other modalities such as chemotherapy or surgery.

### 2.4. Financial Difficulty and Quality of Life

Baseline surveys were completed within 7 days of starting treatment. End of treatment surveys were completed on the last day of radiation therapy. To detail financial difficulty, the European Organisation for Research and Treatment of Cancer (EORTC)-QLQ-C30 survey was used. Question 28 of this questionnaire states “Has your physical condition or medical treatment caused you financial difficulties”, with responses recorded on a Likert-type scale, as follows: “1–Not at all, 2–A little, 3–Quite a bit, 4–Very much”. To assess change in financial difficulty with treatment, we subtracted the baseline score from the score obtained at the end of treatment. For example, with a post-treatment score of 3 and baseline score of 2, the change of financial difficulty would be reported as 1. To determine QOL summary scores, baseline responses to the EORTC-QLQ-C30 were used. Scoring was performed as standard [19]. Summary score was calculated as per the EORTC group [20].

### 2.5. Statistical Analysis

Differences between groups were assessed using the Pearson χ^2^ test for categorical variables and the Wilcoxon test for continuous variables. The Wilcoxon Signed Ranks test was used to compare differences in financial difficulty score, pre- and post-treatment, based on receipt of FC. To examine the change in financial difficulty, the pre-treatment score was subtracted from the post-treatment score. A linear regression model was used to identify variables associated with the change in financial difficulty. The regression coefficients were presented as $\hat{\beta }\pm SE$ (standard error). Those variables with *p*-values < 0.1 were incorporated into a multiple linear regression model. Variables with *p* ≤ 0.05 were considered significant. Statistical analyses were performed using IBM SPSS Version 26 and R 4.0.5.

## 3. Results

Within this cohort of 387 patients, the majority were male (78.0%) and married (60.2%) (Table 1). Most had private health insurance (49.6%) followed by Medicare (42.0%) (Table 1). Nearly half the patients had pharyngeal primaries with 43.9% of all tumors associated with HPV (Table 1). The most common treatment modality was concurrent chemoradiation (62.5%) and volumetric modulated arc therapy (93.5%) (Table 1). Over 90% of the patients were treated in 33–35 fractions (Table 1).

After July 2018, with the hiring of a dedicated financial counselor, the proportion of patients receiving FC increased significantly (5.3% vs. 62.7%, *p* < 0.0001). Patients who underwent FC were more likely to be married or formerly married (*p* = 0.005), to have a lower nodal stage (*p* = 0.006), and to undergo fewer radiation fractions (*p* = 0.002) (Table 1).

Compared with baseline scores, patients who did not undergo FC had a significant increase in reported financial difficulty scores at the end of treatment (*p* = 0.002) (Table 2). On the other hand, there was no difference in pre- and post-treatment scores in patients who had received FC (*p* = 0.588) (Table 2).

Using a linear regression model, each variable was separately assessed for a potential correlation with change in financial difficulty score following treatment (Table 3). The analysis identified female gender with a lower increase in financial difficulty (β = −0.23 ± 0.1, *p* = 0.02). On the other hand, higher nodal positivity (*p* < 0.05) was found to be associated with a larger increase in financial difficulty scores. The univariate analysis also reveals a trend for FC to reduce the increase in financial difficulty, which is marginally significant (β = −0.18 ± 0.094, *p* = 0.052) (Table 3).

Adjusting for gender and nodal status with a multiple linear regression model, FC was significantly associated with change in financial difficulty (β = −0.204 ± 0.096, *p* = 0.035) (Table 4). Therefore, patients who received FC had a 0.2 units lower change in financial difficulty score on average, compared with those with the same gender and nodal stage but without FC.

## 4. Discussion

In this study, unsurprisingly, routine utilization of financial counselor was associated with a significant increase in the number of patients receiving FC (5.3% vs. 62.7%, *p* < 0.0001). Financial difficulty scores at the end of treatment, as compared with baseline, were significantly higher in patients without FC (*p* = 0.002), but not in those with FC (*p* = 0.588). The multiple linear regression analysis showed a significant effect of FC on the change in financial difficulty scores after adjusting for gender and nodal status. The result suggests that, for patients of the same gender and nodal status, receiving FC will reduce the change in financial difficulty score by 0.2 units, on average.

FT is associated with the decline of QOL as well as increased mortality, possibly through a hesitancy to pursue additional treatment or non-compliance [9,10,11,21,22,23].

Despite numerous calls to reduce healthcare costs in the United States, this extremely complex issue will require a multifaceted approach to correct and is likely to worsen in the short term [4,24,25]. One solution to reduce FT is to consider the clinical benefit of new interventions not just against adverse reactions, but also to consider the cost and potential for FT [24]. Current studies of de-escalation of treatment in HNC do factor cost-savings as well [26].

FC provides transparency and clarity for a complex medical system during a stressful time in patients’ life [4,25,27]. Furthermore, unlike modifications of standard therapeutic regimens, the introduction of non-treatment-based interventions to address FT are unlikely to compromise oncologic outcomes. While some have proposed the utility of FC to ameliorate FT, empiric investigation is typically limited to small pilot studies [27,28,29,30,31].

Even though FC was possible, the presence of a full-time employee dedicated to this task and screening all outpatients was clearly beneficial as the proportion of patients receiving FC increased significantly (5.3% vs. 62.7%). As seen with a previous study, awareness, ease of access, and availability are critical to helping overcome barriers preventing from patients receiving FC [27].

Interestingly, while patients who underwent FC were more likely to be married or formerly married and treated with fewer fractions, these factors were not associated with financial difficulty in the regression model. As patients who underwent FC were more likely to have a lower nodal stage, it is vital to account for this association, as increased nodal stage was associated with a greater difference between post-treatment and pre-treatment financial difficulty scores. As the use of multiple treatment modalities was not associated with change in financial difficulty scores, the mechanism behind the association between nodal status and FT is unclear.

In contrast to our findings, randomization to FC was not associated with reduced FT in a previous 95-patient randomized study of patients with metastatic gastrointestinal or lung cancer planned for chemotherapy, who were randomly assigned to receive FC versus no intervention [27]. Notably, only 30% of patients randomized to FC actually completed the full, two-part intervention (in-person and phone counseling) [27]. While another pilot study in non-metastatic solid tumor patients also had difficulty with compliance (59%), financial navigation was association with a reduction in high levels of financial concern [28]. Moreover, in a non-cancer population, the implementation of financial navigation strategies improved patient satisfaction care and cost-concerns [29]. Interestingly, when caregivers were also included in financial navigation, participation rate increased to 78%, suggesting targeting the patient-caregiver dyad is a more effective strategy to implement FC [30].

A limitation to the current study is the use of a single-item question within the EORTC-QLQ-C30 to assess financial difficulty, whereas others have proposed more comprehensive questionnaires including the comprehensive score for financial toxicity (COST), Personal Financial Wellness Scale, the Patient Self-Administered Financial Effects (P-SAFE), as well as an HNC specific survey, the Financial index of Toxicity (FIT) [31,32,33,34]. Furthermore, the causes and consequences of financial difficulty are not characterized in this study. While plans to incorporate a more detailed measure in our clinical evaluation are underway, the single-item financial difficulty question may have use as a screening item to identify those who would benefit from additional financial information.

Additional limitations include potential selection bias in the patients who received FC versus those who did not. Moreover, this analysis was unable to account for median income, employment status, and education level among patients.

Finally, during this study period, no other department at our institution employed a dedicated financial counselor. However, based in part on the findings of this manuscript, our institution plans to implement financial counseling in different clinics as part of a project sponsored by the National Institutes of Health.

## 5. Conclusions

Providing a dedicated financial counselor significantly increased the proportion of HNC receiving FC, resulting in the stabilization of financial difficulty scores post-treatment. Based on a multiple linear regression model, FC was independently associated with reduced financial difficulty. The employment of a financial counselor may be a viable, hospital-based approach to begin to address FT in HNC.

## Figures and Tables

**Table 1:** Patient demographics.

Patient Demographics		All Patients (*n* = 387)			Financial Counseling						
					No (*n* = 285)			Yes (*n* = 102)			
		Median (IQR)	*n*	%	Median (IQR)	*n*	%	Median (IQR)	*n*	%	*p*-Value
Age (Years)		62 (55.4–68.5)			62 (55.5–68.5)			62 (55–69)			0.717
Gender	Male		302	78.0%		225	78.9%		77	75.5%	0.469
	Female		85	22.0%		60	21.1%		25	24.5%	
Marital status	Single		73	18.9%		67	23.5%		6	5.9%	0.005
	Married		233	60.2%		163	57.2%		70	68.6%	
	Divorced		48	12.4%		30	10.5%		18	17.6%	
	Widowed		23	5.9%		16	5.6%		7	6.9%	
	Unknown		8	2.1%		7	2.5%		1	1.0%	
	Separated		1	0.3%		1	0.4%		0	0.0%	
	Life partner		1	0.3%		1	0.4%		0	0.0%	
Insurance	Private		182	49.6%		131	48.7%		51	52.0%	0.545
	Medicare		154	42.0%		117	43.5%		37	37.8%	
	Medicaid		31	8.4%		21	7.8%		10	10.2%	
Financial assistance	No		246	63.6%		175	61.4%		71	69.6%	0.321
	Yes		29	7.5%		22	7.7%		7	6.9%	
	Missing		112	28.9%		88	30.9%		24	23.5%	
Pharynx	Non-pharynx		183	47.3%		136	47.7%		47	46.1%	0.776
	Pharynx		204	52.7%		149	52.3%		55	53.9%	
HPV status	Negative		217	56.1%		156	54.7%		61	59.8%	0.376
	Positive		170	43.9%		129	45.3%		41	40.2%	
T stage	T0		29	7.6%		25	8.9%		5	4.9%	0.437
	T1		55	14.3%		40	14.2%		15	14.7%	
	T2		112	29.2%		86	30.5%		26	25.5%	
	T3		116	30.2%		83	29.4%		32	31.4%	
	T4		72	18.8%		48	17.0%		24	23.5%	
N stage	N0		83	21.6%		60	21.3%		23	22.5%	0.006
	N1		90	23.4%		55	19.5%		35	34.3%	
	N2		174	45.3%		141	50.0%		33	32.4%	
	N3		37	9.6%		26	9.2%		11	10.8%	
Treatment	RT		28	7.2%		18	6.3%		10	9.8%	0.462
	Surgery + RT		45	11.6%		31	10.9%		14	13.7%	
	ChemoRT		242	62.5%		184	64.6%		58	56.9%	
	Surgery + ChemoRT		72	18.6%		52	18.2%		20	19.6%	
Technique	VMAT		362	93.5%		271	95.1%		91	89.2%	0.38
	3DCRT		25	6.5%		14	4.9%		11	10.8%	
Fractions	33–35		355	91.7%		269	94.4%		86	84.3%	0.002
	28–30		32	8.3%		16	5.6%		16	15.7%	
QOL summary score		81.6 (72.4–87.8)			82.3 (72.6–88.0)			79.4 (71.1–86.3)			0.13

IQR: interquartile range; HPV: human papilloma virus; RT: radiation therapy; ChemoRT: concurrent chemoradiation; VMAT: volumetric modulated arc therapy; 3DCRT: 3-dimensional conformal radiation therapy; QOL: quality of life.

**Table 2:** Financial difficulty scores by receipt of financial counseling.

EORTC QLQ-C30 Question 28	Financial Counseling							
	No (*n* = 285)				Yes (*n* = 102)			
	Pre		Post		Pre		Post	
Financial Difficulty	*n*	%	*n*	%	*n*	%	*n*	%
1—Not at all	172	60.4%	150	52.6%	60	58.8%	59	57.8%
2—A little	69	24.2%	73	25.6%	21	20.6%	25	24.5%
3—Quite a bit	31	10.9%	45	15.8%	16	15.7%	14	13.7%
4—Very much	13	4.6%	17	6.0%	5	4.9%	4	3.9%

Pre: pre-treatment; Post: post-treatment.

**Table 3:** Correlation with change in financial difficulty by linear regression.

Variable	β	SE	*p*-Value
Female	−0.23	0.1	0.0242
Married	−0.07	0.085	0.3971
Financial Counseling	−0.18	0.0941	0.0515
Insurance			
Private	reference		
Medicare	0.01	0.091	0.8694
Medicaid	0.21	0.1616	0.199
Financial Assistance	−0.17	0.1613	0.2913
Age (>60 years)	0.01	0.0846	0.9047
Pharynx primary	0.04	0.0834	0.6741
T stage			
T0	reference		
T1	−0.1	0.189	0.605
T2	−0.03	0.1716	0.869
T3	−0.14	0.171	0.4203
T4	−0.19	0.1811	0.2872
N stage			
N0	reference		
N1	0.25	0.1242	0.0425
N2	0.26	0.1089	0.016
N3	0.38	0.1614	0.0195
HPV positive	0.04	0.0839	0.6183
Treatment			
RT	reference		
Surgery + RT	−0.01	0.1969	0.9454
ChemoRT	0.05	0.1633	0.774
Surgery + ChemoRT	0.23	0.1822	0.2112
3DCRT	0.02	0.1695	0.9294
Fractions (28–30)	−0.08	0.1512	0.5906
QOL summary score	−0.001	0.003	0.754

SE: standard error; HPV: human papilloma virus; RT: radiation therapy; ChemoRT: concurrent chemoradiation; 3DCRT: 3-dimensional conformal radiation therapy; QOL: quality of life.

**Table 4:** Multiple linear regression model for change in financial difficulty.

Variable	β	SE	*p*-Value
Female	−0.2068	0.0999	0.0391
N stage			
N0	reference		
N1	0.2783	0.1247	0.0263
N2	0.2314	0.1085	0.0336
N3	0.3695	0.1604	0.0218
Financial Counseling	−0.2039	0.0964	0.0351

SE: standard error; F-test of overall significance: *p* = 0.0054; R^2^ = 0.04.

## Data Availability

Farrugia, Yu, and Singh had full access to all the data in the study and take responsibility for the integrity of the data and the accuracy of the data analysis. The data underlying this article cannot be shared publicly for the privacy of individuals that participated in the study. The data are available from the corresponding author upon reasonable request.

## Supplementary Materials

The following are available online at https://www.mdpi.com/article/10.3390/cancers13112516/s1, Supplemental document 1: understanding your health insurance benefits.
