# Supplementary material for: Financial Counseling Is Associated with Reduced Financial Difficulty Scores in Head and Neck Cancer Patients Treated with Radiation Therapy
Source: Cancers (Basel). 2021 May 21;13(11):2516. doi: 10.3390/cancers13112516 (PMC8196601; doi:10.3390/cancers13112516)
Supplement: Supplementary file 1 [file cancers-13-02516-s001.zip › cancers-1232848-supplementary.pdf]

ROSWELL PARK COMPREHENSIVE CANCER CENTER

UNDERSTANDING YOUR HEALTH INSURANCE BENEFITS

RADIATION THERAPY

Name: **Attending MD:**
Date of Review: **MR#:**

Thank you for choosing Roswell Park Comprehensive Cancer Center to provide your radiation therapy treatment(s). We understand this can be a challenging time in your life, and that beginning a course of treatment can increase anxiety and concerns about the unknown, including your financial responsibility for treatment. We are here to help alleviate your concerns and provide you with information to help you understand the financial aspect of treatment. We have a Financial Counselor who will assist you in understanding your benefits and offer various options available to you to help manage your healthcare expenses. If you have any questions regarding the information below, please contact , Financial Counselor, at or you may request to meet her at any time along your course of treatment.

Each individual's benefits are different, but there are common terms that may apply. The following information is prepared to assist you in understanding your insurance benefits pertaining to radiation therapy. This is not a guarantee of benefits; however we have made every effort to obtain correct benefit information from your insurance carrier. Final determination of your radiation therapy benefits will be dictated by your insurance company at the time the claim for services is processed. Therefore, your financial responsibility may be adjusted to reflect this.

☐ ***ANNUAL DEDUCTIBLE*** This is the amount of annual out-of-pocket expense for covered medical services your insurance requires you to pay each year to a health care provider before the insurance begins paying for services.

☐ ***AMOUNT met to date*** (as of the most current information available)

☐ ***BALANCE*** of the Annual Deductible for this calendar year (as of the most current information available)

☐ ***OUT-OF-POCKET MAXIMUM***: This is the amount of co-insurance that you are required to pay annually. Co-pays may/may not apply to this amount, as it varies by insurance plan.

Do my co-pays apply to my out-of-pocket maximum? ☐ Y ☐ N
Does my deductible apply to my out-of-pocket maximum? ☐ Y ☐ N

☐ ***OUT-OF-POCKET MAXIMUM MET TO DATE*** *as of the most current information

Most plans are structured based on Co- insurance or Co-pays. Your plan requires you to pay a:

☐ ***CO-INSURANCE:*** This is the percentage of the visit or treatment that your insurance requires you to pay. Your deductible must be met before your insurance begins to pay their portion. Each course of therapy is unique to patient circumstances, however, we have used the information available to us to estimate the services you may require. Based on your insurance plan's contract with the Roswell Park, your estimated financial responsibility for radiation therapy will be

☐ **DAILY CO-PAYMENT:** This is the amount that you pay for each radiation treatment. Since this is a specialist office, the amount may be higher than what you pay for other types of healthcare visits. Each course of therapy is unique to patient circumstances. We have used the information available to us to estimate the services you may require. Based on your insurance contract, we expect your copay responsibility for the anticipated course of therapy to be approximately

Co-payments for daily treatments will be collected weekly on Fridays, so please stop at the reception desk to pay your weekly co-payment.

OTHER IMPORTANT INFORMATION:

- *Charges* for some radiation services will occur on dates that you are not here. Some services are performed between visits to develop your radiation treatment plan in preparation for your course of treatment.
- *Pre Authorization* may be required by your insurance company. *IF your insurance requires a pre- authorization* for treatment, Roswell staff will obtain any necessary authorization on your behalf. Depending on the pre-auth benefit management company, you may receive mail and/or calls with this information.
